# Fermented By-Products of Banana Wine Production Improve Slaughter Performance, Meat Quality, and Flavor Fingerprint of Domestic Chicken

**DOI:** 10.3390/foods13213441

**Published:** 2024-10-28

**Authors:** Zhichun Li, Xuemei He, Yayuan Tang, Ping Yi, Ying Yang, Jiemin Li, Dongning Ling, Bojie Chen, Hock Eng Khoo, Jian Sun

**Affiliations:** 1 Agro-Food Science and Technology Research Institute, Guangxi Academy of Agricultural Sciences, Nanning 530007, China; hexuemei@gxaas.net (X.H.); tangyayuan@gxaas.net (Y.T.); pingyi@gxaas.net (P.Y.); yangying@gxaas.net (Y.Y.); lijiemin@gxaas.net (J.L.); lingdongning@gxaas.net (D.L.); jiansun@gxaas.net (J.S.); 2 Guangxi Key Laboratory of Fruits and Vegetables Storage-Processing Technology, Nanning 530007, China; 3 College of Chemistry and Bioengineering, Guilin University of Technology, Guilin 541006, China; bjchen@stu.scu.edu.cn (B.C.); 2020153@glut.edu.cn (H.E.K.); 4 College of Biomass Science and Engineering, Sichuan University, Chengdu 610065, China

**Keywords:** aroma, fat composition, sensory analysis, shear force, water-holding capacity

## Abstract

This study aimed to compare the effects of incorporating fermented feed into daily diets on the slaughter performance, meat quality, and flavor compounds of 120 domestic chickens over a 140-day period. A total of five groups (n = 24), including the control group (CK) of the Guangxi Partridge chickens received a standard base diet. The other four groups were provided with pellets that had been added with 10% fermented banana peel (Pe-10), 20% fermented banana peel (Pe-20), 10% fermented banana pulp residue (Pu-10), and 20% fermented banana pulp residue (Pu-20). The flavor compounds in the meat samples of the chickens in these groups were determined using the gas chromatographic method. The results demonstrated that the chickens in the Pe-10, Pe-20, Pu-10, and Pu-20 groups exhibited pectoral muscle percentages, thigh muscle percentages, and total fatty acid content of chest meat that were higher than those observed in the CK group. The moisture content, meat color, carcass weight, total net weight, and abdominal fat percentage of the meat samples in these experimental groups exhibited no notable differences. The flavor compounds in the meat samples of the chickens fed with the two concentrations of fermented banana peel and banana residue were found to be significantly different from those in the control group, with *p*-values less than 0.05. As the quantity of fermented banana peel incorporated into the daily ration was increased from 10% to 20%, a notable alteration in the flavor compounds present in the chicken samples was observed. The chickens that were provided with fermented banana peels and pulps in their diets exhibited superior slaughter performance and meat quality, particularly in the case of the Pu-10 group, in comparison to the control chickens.

## 1. Introduction

Bananas are the fourth most widely cultivated fruit in the world, with the majority of global production occurring in southern China. As indicated in the China Statistical Yearbook 2021, China produced 11.513 million tons of bananas in 2020 [1], with banana peels accounting for 30–40% of the total yield [2]. The large-scale disposal of these banana peels may give rise to a number of environmental concerns, including water and air pollution. Banana peel and its residue contain nutrients and phytochemicals [3,4]. Banana peel is a rich source of dietary fiber [5] and pectin [6]. These banana by-products have been developed into animal feed [7]. The existing literature indicates the utilization of banana peels in ruminant feeds [8]. Furthermore, banana waste devoid of functional properties is typically discarded, incinerated, or processed into fertilizer.

The microbial fermentation of banana peels has the potential to enhance their palatability and nutritional value. The scientific literature indicates that the Lohmann broiler chickens fed with fermented banana peels from day 22 to day 38 exhibited superior slaughter performance and meat quality in comparison to the chickens in the control group [9]. The findings revealed that the chickens that were fed with the fermented banana peels exhibited enhanced weight gain and an improvement in their lipid profile. Moreover, the breast meat of the supplemented chickens demonstrated a higher lightness value than that of the meat samples in the control group. Additionally, another study indicated that the growth performance of the male native chickens fed with the fermented banana peels for a period of ten weeks exhibited positive outcomes [10]. In a related study, Caicedo [11] investigated the impact of incorporating natural yogurt, whey, and molasses into banana silage fermentation. The physicochemical, biological, and sensory indexes of these fermented banana silages were determined, and the results demonstrated that these silages were suitable for pig breeding. The experimental group of Siamese catfish, which was fed a diet containing 20% fermented banana peel, exhibited comparable feed consumption, specific growth rate, feed efficiency, fat retention, and energy retention to the control group, which was fed a commercial diet [12].

The fermented by-products of ripe bananas, including banana peels and pulp residue collected from the wine fermentation process, represent a valuable source of waste with potential functional applications. The presence of protein, polysaccharides, and dietary fibers in the by-products renders them suitable for use as ingredients in the production of animal feeds, as evidenced by the literature [13]. The extant scientific literature evinces the salutary effects of fruit peels on the slaughter performance and meat quality of poultry [14,15]. However, there is a dearth of studies examining the impact of incorporating fermented banana pulp residue into poultry diets, particularly with regard to the effects on feed quality and the flavor components of poultry meat. No comparison has been made between the nutritional parameters of meat from chickens fed fermented and unfermented fruit peels.

It is hypothesized that the incorporation of fermented by-products, including banana peels and banana pulp residues obtained from banana wine production, into poultry diets will result in improvements in the slaughter performance and meat quality of chicken fed with the poultry diet added with the fermented by-products. The objective of this study was to evaluate the effects of incorporating fermented banana peels and pulp residues into poultry diets on slaughter performance, meat quality, and flavor components. This study makes a contribution to the existing literature by demonstrating the potential of banana by-products from wine production as a source of poultry feed to improve the slaughter performance and meat quality of domestic chicken in Guangxi.

## 2. Materials and Methods

### 2.1. Fermented Banana Peel and Banana Residue

The banana variety utilized in this experiment was Guijiao 6 (Musa AAA). It is the primary cultivar grown in the Guangxi region. The fruit was cultivated in a local plantation in the Guangxi region. The harvested unripe bananas were stored at room temperature in the laboratory prior to undergoing further processing. The ripened fruit peel was separated and allowed to ripen. Subsequently, the pieces were reduced in size and inoculated with a fermentation agent. The banana peels were fermented with the use of yeast, emulating the procedures employed in wine production, albeit with certain modifications [16]. Following homogenization, the fruit peels were packed, sealed, and then stored at a room temperature of 25 °C for a period of 15 days. Following partial fermentation, 0.8% pectinase and 0.5% cellulase were added to them, and they were left to stand for 2 h. The enzymatically hydrolyzed peel samples were filtered, and the filtrate was collected. In contrast, the pulp residues were inoculated with the fermentation agent, packed, and sealed before undergoing fermentation at room temperature for 15 days.

The ash content (%) of the fermented banana samples was determined by ashing the oven-dried banana samples at 550 °C for 3 h, while the dry matter content was determined by oven-drying the samples at 105 °C until a constant weight was obtained, as previously described in the literature [17]. The carbohydrate content was estimated using the anthrone-sulfuric acid method [18], and the crude protein content was determined using the Kjeldahl method, as outlined in the literature [19]. The pH values of the sample homogenates (1:9, *w*/*v*, diluted with normal saline) were determined using a PHS-3C benchtop pH meter (INESA Scientific Instrument Co., Ltd., Shanghai, China). The titratable acidity was subsequently calculated by titrating with a titration-calibrated sodium hydroxide solution. The number of lactic acid bacteria (LAB) present in the sample homogenates was determined using the standard plate count method [20]. The determinations of these chemical constituents in the fermented banana samples were conducted in triplicate. The chemical constituents of the fermented samples are presented in Table 1.

### 2.2. Poultry Feeding Experiment

The poultry experimentation was conducted in accordance with the ethical standards set forth by the Animal Care and Use Committee of Guangxi Academy of Agricultural Sciences, Nanning, China (GXAAS/AEEIF/005). Prior to the commencement of the feeding experiment, 120 birds of the Guangxi Partridge hens (*Gallus gallus domesticus*) were acclimatized at the Tiandong poultry farm in Guangxi. The hens were of a crossbreed genetic lineage. The hens were 12 weeks old at the outset of the study, with an average body weight of 1.25 ± 0.02 kg. The subjects were randomly assigned to one of five experimental groups (n = 24), including a control group (CK) and four supplementation groups. The experiment was conducted over a period of 140 days.

All experimental chickens were provided with unrestricted access to the experimental feeds and tap water for a period of 140 days. Twenty-four domestic chickens were randomly assigned to one of five groups, comprising a control (CK) group and four supplementation groups. The CK group was fed a basal diet, while the other four supplementation groups were fed diets supplemented with 10% fermented banana peel (PE-10), 20% fermented banana peel (PE-20), 10% fermented banana pulp residue (PU-10), and 20% fermented banana pulp residue (PU-20), respectively. During the experimental period, all chickens had access to tap water ad libitum. The poultry house was cleaned on a weekly basis and ventilated with ambient temperatures that ranged from 27 °C to 33 °C.

The composition of the poultry feed is presented in Table 2. The basal diet consisted of a corn and soybean mixed meal. The feed consisted of the following ingredients: 57.5% corn, 25% soybean meal, 5% wheat bran, 2.5% soy oil, and 10% premix. The feed contained 17% crude protein. The two essential amino acids were lysine (0.8%) and methionine (0.4%). The energy content was 2.75 megacalories per kilogram (MC/kg).

### 2.3. Preparation of Animal Samples

At the conclusion of the feeding trial (32 weeks of age), all chickens that had been fasted were subjected to a blood draw prior to slaughter. The poultry was fasted for 12 h prior to being sacrificed in accordance with the procedures outlined in the GB/T 19478-2018 procedure [21]. Following exsanguination via the jugular vein, the carcass was blanched in boiling water, and the feathers were removed by pulling them off the body. The weights of all organs were recorded, and the pectoral and thigh muscles were collected for subsequent analysis. Additionally, the layers of abdominal fat surrounding the muscular stomach and abdomen of each hen were separated and weighed. The pectoral muscles, situated along the sternum ridge, were obtained with the chicken skin removed. The thigh muscles were collected after the skin, subcutaneous fat, and bones were removed. The slaughter performance of the chickens was presented in the form of the carcass weights and percentages of pectoral and thigh muscles. A single sample replicate of the aforementioned experimental parameters was determined for each experimental hen, with a total of 24 birds comprising each experimental group.

### 2.4. Nutritional Composition and Color Values of Chicken Chest Meats

The pectoral muscle, commonly referred to as “chest” or “breast” meat, was analyzed for its moisture content and fatty acid (FA) composition. These parameters were analyzed in accordance with the Chinese standard analytical methods. The standards in question were GB 5009.3-2016 [22] and GB 5009.168-2016 [23], respectively. The L*, a*, and b* values were analyzed at the 45-min mark post-slaughter. The values were determined using a chromometer (CR400/410, Minolta, Osaka, Japan) [24]. As with the parameters pertaining to slaughter performance, a single sample replicate was determined for each of the 24 chickens in each experimental group.

### 2.5. Shear Force and Water-Holding Capacity of Chicken Chest Meats

The shear force test and water loss analysis were conducted on the chest meat samples within 24 h of slaughter. The shear force value of the meat samples was determined in accordance with the methodology outlined in the referenced literature [25]. The meat samples were initially subjected to a water bath maintained at a constant temperature of 80 °C. The meat samples were cut into cubes measuring 2.5 cm in length, 1.0 cm in width, and 0.25 cm in height. The RH-N50 Meat Tenderness Tester (Runhu Instrument Co., Ltd., Guangzhou, China) was employed to ascertain the shear force value at three distinct points, with the resulting values averaged. The water-holding capacity of the meat samples was determined in accordance with the methodology described by Zhang et al. [26] using an RH-1000 Water Holding Capacity Tester (Runhu Instrument Co., Ltd., Guangzhou, China). In summary, the meat sample was subjected to a pressure of 35 kg for a period of 5 min, after which it was weighed. The shear force test and water-holding capacity of the meat samples were determined for each individual hen in each experimental group using a single sample replicate.

### 2.6. Determination of Flavor Compounds in Chicken Chest Meats

The analysis was conducted using gas chromatography-ion mobility spectrometry (GC-IMS). The GC analysis of flavor substances was conducted using the method described in the literature with some modifications [27]. In summary, 2.0 g of chest meat samples were weighed and placed in a 20 mL headspace bottle, incubated at 90 °C for 15 min, and analyzed by a FlavourSpec^®^ GC-IMS flavor analyzer (Dortmund, Germany). The following analytical conditions were employed for the GC-IMS. The stationary phase was an FS-SE-54-CB-1 capillary column (15 m, ID: 0.53 mm), the column temperature was 60 °C, the IMS temperature was 45 °C, the injection volume was 500 μL, high-purity nitrogen was used as the carrier gas, and the total run time was 30 min. The gradient gas flow conditions were as follows: 0–2 min, 2 mL/min; 2–10 min, 2–10 mL/min; 10–20 min, 10–100 mL/min; 20–30 min, 100–150 mL/min. A pooled sample of each experimental group was subjected to GC-IMS analysis in five replicates.

### 2.7. Statistical Analysis

The statistical analysis was conducted using SPSS (version 20.0, SPSS, Inc., Chicago, IL, USA). The data normality was evaluated using the detrended Q-Q plot, which revealed that most of the data were normally distributed, with the exception of the analysis of flavor components. The data were analyzed using one-way ANOVA coupled with Duncan’s multiple-range test. A *p*-value of less than 0.05 was considered statistically significant, indicating a notable distinction between the groups (n = 24 for each group). The qualitative, quantitative, and fingerprint characteristics of the flavor compounds in the meat samples were determined using the VOCal, Reporter, Gallery Plot, and Dynamic PCA plug-ins. The SIMCA method was employed for the analysis of flavor compounds, utilizing the orthogonal partial least squares approach and cluster analysis.

## 3. Results and Discussion

### 3.1. Slaughter Performance

The slaughter performance of poultry is an important quality indicator in the context of poultry farming. The provision of different standard daily feeds to poultry has been demonstrated to promote optimum growth [28]. The data presented herewith pertain to the slaughter performance of the chickens in question and include carcass weight, abdominal fat, and percentages of chicken muscles. The mean weight gain of the experimental chickens at the conclusion of the study period ranged from 2.51 kg to 2.83 kg. The weight gain of all supplementary groups was not found to be significantly higher than that of the control group (*p* > 0.05). The findings on weight gain were corroborated by a previous study in which broilers fed fermented banana peels exhibited no significant change in the final body weight [29]. A review of the scientific literature reveals that broiler chickens fed diets supplemented with various fruit peels or rinds demonstrated enhanced growth performance, particularly in terms of increased final body weight [20]. In contrast, the broiler chicken fed with the fermented orange peel exhibited a reduction in final weight gain [30].

The addition of the fermented banana peels and pulp residues to the diet of the experimental chicken promoted growth, as evidenced by the data presented in Table 3. The results demonstrated that the pectoral muscle percentages of the chickens in the supplementation groups were significantly higher than those of the CK group following the addition of varying proportions of fermented banana peel and banana residue to their daily feed (*p* < 0.05), with the exception of the Pu-20 group. The percentages of the thigh muscle of chicken in the Pe groups were notably higher than in the control group (*p* < 0.05). No significant differences were observed in the remaining tested parameters of the chickens in the supplementation groups when compared with the control group (*p* > 0.05), with the exception of the carcass weight and abdominal fat content of the chickens in the Pe-10 and Pu-10 groups, respectively.

The existing literature indicates that the inclusion of the by-product of fermented fruit or fruit waste in poultry feeds may enhance the slaughter performance of chickens and the quality of their meat [31]. The slaughter performance of chickens fed a diet containing fermented grape skin was superior to that of the control group. However, no statistically significant differences were observed between the fermented and unfermented sample groups (*p* > 0.05) [32]. Another study demonstrated that the incorporation of pomegranate peel powder into broiler diets resulted in enhanced slaughter performance relative to the control group [33]. The broiler chickens fed with the highest dosage of pomegranate peel powder (4 g/kg diet) exhibited a significantly higher percentage of breast meat than the groups receiving 2 and 3 g/kg diet and the control group (without additive) (*p* < 0.05). In contrast, the addition of either 3% unfermented dried apple or 3% unfermented dried cherry pomace to the diet of broiler chickens had no discernible impact on meat quality, with the exception of the broiler chickens fed with unfermented dried strawberry pomace [34]. The results indicated that the broiler chickens fed a diet supplemented with dried strawberry pomace exhibited a reduction in their final body weights. The aforementioned studies corroborate our findings that the inclusion of fermented fruit peels and by-products in poultry diets has the potential to enhance both slaughter performance and meat quality.

### 3.2. Quality Parameters of Chicken Chest Meats

#### 3.2.1. Meat Brightness, Colors, and Tenderness

The primary quality characteristics of the chicken chest samples are presented in Table 4. The color of the meat is a primary indicator of its quality. The parameters include brightness (L*), redness (a*), and yellowness (b*). The results indicate that there are statistically significant differences in the L*, a*, and b* values among the experimental groups (*p* < 0.05). The meat samples of the chicken in the Pu-20 group exhibit the most notable increase in the L* value (*p* < 0.05), while the L* values of the other experimental chicken meats do not demonstrate a significant elevation (*p* > 0.05). However, an increase in meat redness is observed for the groups that received diets supplemented with different fermented samples in comparison to the control (CK) group. The meat sample from the Pu-10 group exhibited a darker hue than that of the control group.

The a* value of the meat samples was found to be significantly higher in all supplemented groups in comparison to the control group (*p* < 0.05), with the exception of the Pu-20 group. With the exception of the Pe-20 and Pu-10 groups, the meat samples in the supplementary groups exhibited a significantly lower b* value in comparison to the control group. Additionally, the meat samples in the Pe-10 and Pu-10 groups exhibited the most significantly elevated a* and b* values, respectively (*p* < 0.05). The meat samples in the Pu-20 group exhibited low a* and b* values despite displaying the highest L* value. Furthermore, the meat samples in the Pe-10 group exhibited the lowest b* value and the highest a* value.

The literature indicates that an elevated a* value indicates a higher red hue, whereas a higher b* value indicates a higher yellow hue [35]. The chest meat samples of the chicken in the Pe-10 group exhibited intense redness and minimal yellow hues with moderate brightness. This suggests that the meat sample from this group exhibited superior meat quality compared to the other groups. The low meat redness of the chicken in the CK group demonstrates that the supplementation of fermentation banana samples effectively improved the meat quality. In contrast, a previously published study reported that the breast meat of broilers fed a diet containing 5% to 15% fermented banana peel exhibited a significantly lower a* value (*p* < 0.01) in comparison to the control group [9]. Furthermore, the study reports a markedly elevated L* value (*p* < 0.01) for the breast meat samples in the supplementary groups in comparison to the control group. The experimental chickens that were fed a diet containing dried grapefruit peel exhibited significantly lower L*, a*, and b* values than those of the control chickens (*p* < 0.05). However, this was not observed in the case of the chickens that were fed a diet containing dried orange fruit peel [36].

The observed variation in meat coloration may be attributed to differences in the composition of the animals’ diets. The reduction in meat redness may be attributed to the oxidation of myoglobin [37]. The discoloration of meat is associated with an increase in lipid oxidation, which results in the formation of high ferric myoglobin. The accumulation of mefermyoglobin may be attributed to the decreased activity of mefermyoglobin reductase [38]. The scientific literature indicates that the reduced redness of meat in the CK group may be attributed to the elevated concentration of vitamin E in the fermented banana samples, which subsequently inhibits lipid peroxidation [39]. Other factors, including temperature, oxygen partial pressure, pH value, light, osmotic pressure, and surface microbial activity, may influence the morphology of myoglobin, thereby contributing to the observed color variation [40].

As illustrated in Table 4, the shear forces of the meat samples from the chicken in the supplementation groups were markedly lower than those in the control group (*p* < 0.05). The meat sample of the chicken in the Pe-10 group exhibited the most notable reduction in shear force (*p* < 0.05). The findings of this study indicate that the addition of 10% fermented banana samples to poultry feeds resulted in superior meat quality compared to the inclusion of 20% fermented banana samples. Hashizawa et al. demonstrated that chickens reared at elevated temperatures (30 °C) exhibited reduced shear forces compared to those maintained at lower temperatures (24 °C) [39]. Additionally, the literature indicates that the inclusion of fermented soybean meal in poultry diets enhances the quality of the resulting meat [41].

The tenderness of meat is indicative of its quality [42]. It is typically expressed as a shear force value. The tenderness of meat is contingent upon the extent of proteolysis of myofibrillar protein, which is instrumental in maintaining the structural integrity of muscle fibers [43]. The most effective method for evaluating the tenderness of meat is through the measurement of shear force. The shear force value of chicken chest meat declines with the prolongation of storage time. A high shear force value indicates a reduction in the freshness of the meat sample, which, in turn, results in a reduction in its chewiness. This is due to the inherent tenderness of fresh meat. Accordingly, a higher shear value is indicative of a greater concentration of myofibrillar protein in the chicken chest meat. These findings are corroborated by a previous study in which breast meat from chickens fed a broiler diet supplemented with dried orange peels exhibited a significantly lower shear force value (*p* < 0.05) than that of the control group [36]. In contrast, the addition of pomegranate peel powder to the broiler diet did not result in any statistically significant changes (*p* = 0.10) in the tenderness of the breast meat samples when compared to the control sample [44]. In conclusion, the addition of fermented banana peels to the animal diet has been demonstrated to enhance the quality of the breast meat, particularly in terms of color and tenderness.

#### 3.2.2. Moisture Content and Water-Holding Capacity

The findings indicated that the moisture content of the chest meat samples in the experimental and control groups exceeded 70%. The moisture content of the meat samples in the Pe groups was comparable to that of the CK group, whereas the meat samples in the Pu groups exhibited a significantly lower moisture content than that observed in the CK group (*p* < 0.05). The lowest moisture content was observed in the meat sample from the Pu-20 group. In addition to its low moisture content, the meat sample from the Pu-20 group demonstrated a water-holding capacity that was less than that observed in the other supplementation groups. The highest water-holding capacity was observed in the Pe-10 group.

The addition of varying quantities of fermented banana samples to the poultry feeds did not significantly impact the meat quality, particularly in terms of color, tenderness, and water-holding capacity. The water-holding capacity of meat is defined as the ability of postmortem animal muscles to retain their original moisture under the influence of external forces, which impact the meat’s freshness and tenderness [45]. The meat with a high water-holding capacity was observed to be more tender and juicy. In this study, the meat samples with a high water-holding capacity exhibited a high moisture content. Moreover, the water-holding capacity of chicken meat samples may be correlated with alterations in the L* values [9]. Furthermore, the elevated moisture content of the broiler meat sample can be attributed to the augmented water-holding capacity and protein content of the meat sample [14].

The existing literature indicates that the addition of 2 g of non-fermented pomegranate peel powder to a kg of broiler diet has been predicted to enhance the water-holding capacity of the breast meat of the broiler chickens [44]. The enhanced water retention capacity may be attributed to the reduced cooking loss observed in the meat sample. The incorporation of hydroethanolic guava peel extract into broiler diets resulted in a slight enhancement in the water-holding capacity of the breast meat of the diet-fed broiler chicken, particularly in the case of broiler diets with moderate amounts of the peel extract [46]. Furthermore, the literature indicates that poultry fed with probiotics exhibits enhanced water-holding capacity in its meat, particularly when supplemented with fermented fruit peels or by-products [47]. It can be concluded that chickens fed a diet containing a moderate concentration of fermented fruit by-products will exhibit an improvement in the water-holding capacity of the meat samples.

### 3.3. Fatty Acid Composition of Chicken Chest Meats

The composition of fatty acids (FAs) in the chest meat samples from the experimental groups is presented in Table 5. The results demonstrate that 12, 19, 22, 13, and 15 FAs were identified in the meat samples of the CK, Pe-10, Pe-20, Pu-10, and Pu-20 groups, respectively. The identified FAs were classified as saturated fatty acids (SFAs), monounsaturated fatty acids (MUFAs), and polyunsaturated fatty acids (PUFAs). The meat samples from the Pe groups exhibited the highest total FA content, followed by those from the Pu and CK groups. However, no statistically significant differences were observed in the total FA content between these experimental groups (*p* > 0.05), with the exception of the control and Pu-10 groups. The meat sample in the CK group exhibited the lowest total SFA content, followed by the Pu and Pe groups. The meat sample in the Pu-20 group exhibited the highest total PUFA content in comparison to the other experimental groups, although this was not the case for the total MUFA content. The content of these FAs, including total SFAs, total MUFAs, and total PUFAs, was not significantly different between the experimental groups, with the exception of the control group (*p* > 0.05).

The SFAs are primarily composed of palmitic acid (C16:0) and stearic acid (C18:0). The predominant MUFA in the meat samples was oleic acid (C18:1n9c), while the predominant PUFA was linoleic acid (C18:2n6c). These findings are consistent with those reported in the literature [36]. The highest concentrations of palmitic, stearic, oleic, and linoleic acids were observed in the meat samples from the Pe-10, Pe-20, Pe-20, and Pe-20 groups, respectively. The levels of these FAs in all supplementation groups were higher than in the control group, with the exception of the Pu-10 group. Additionally, the oleic acid content in the Pe groups was observed to be higher than that in the Pu groups. Conversely, the trans-oleic acid (C18:1n9t, elaidic acid) was identified in all meat samples, whereas only the meat sample in the CK group exhibited trans-linoleic acid (C18:2n6t, linolelaidic acid). The meat samples from chickens that had been fed the Pe samples exhibited a higher trans-oleic acid content than those from chickens that had been fed the Pu samples. The lowest concentration of trans-oleic acid was observed in the meat sample from the Pu-10 group.

Despite the meat sample in Pe-20 exhibiting the highest total FAs content, pentadecanoic acid (C15:0), docosanoic acid (C22:0, behenic acid), and cis-15-tetracosenoic acid (C24:1, nervonic acid) were not identified in this particular meat sample. Pentadecanoic acid is a FA that is typically found in eggs, milk, poultry, and ruminant meats. In this study, pentadecanoic acid was identified exclusively in the meat sample of the Pe-10 group, while nervonic acid was detected in both Pe-10 and Pu-20. Undecanoic acid (C11:0), tridecanoic acid (C13:0), and icosanoic acid (C20:0) were exclusively identified in the meat samples of the Pe-20 group. Additionally, medium-chain FAs were not identified in the chicken fed with the fermented banana pulp residue (Pu-10 and Pu-20 groups) and the control group. Among the long-chain FAs, C15:0 and C20:0 were not detected in the Pu-10, Pu-20, and CK groups.

It is possible that the trans-FAs present in the chest meat samples may have been introduced during the de-feathering process, which involved blanching the meat in boiling water. The heating of unsaturated FA-containing chicken muscles resulted in the oxidation of FAs [48]. The application of elevated temperatures may facilitate the release of these volatiles [49]. Therefore, it can be concluded that the oxidation of FAs occurred in the meat samples, resulting in an increase in the levels of flavor substances. It is possible that this FA oxidation may result in alterations to the flavor of the meat [50]. The elevated levels of trans-fat identified in the meat samples of the Pe groups may be attributed to the antioxidant content of banana peel being comparatively lower than that of banana pulp. The ingestion of antioxidant-rich feeds by the broiler chicken may also assist in maintaining oxidative stability in their muscles [51]. Consequently, the oxidation of FAs in skeletal muscles is reduced. The findings of this study are corroborated by the literature, which indicates that rabbits fed an antioxidant-rich extract exhibited lower trans-FA levels than the control group [52].

### 3.4. Flavor Compounds of Chicken Chest Meats

**Figure 1:**
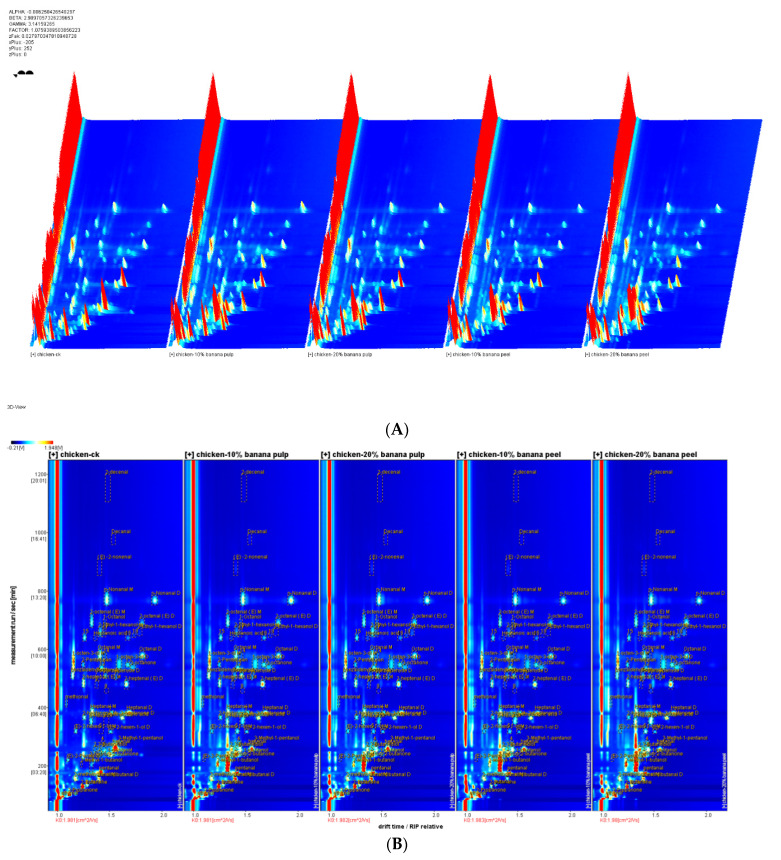

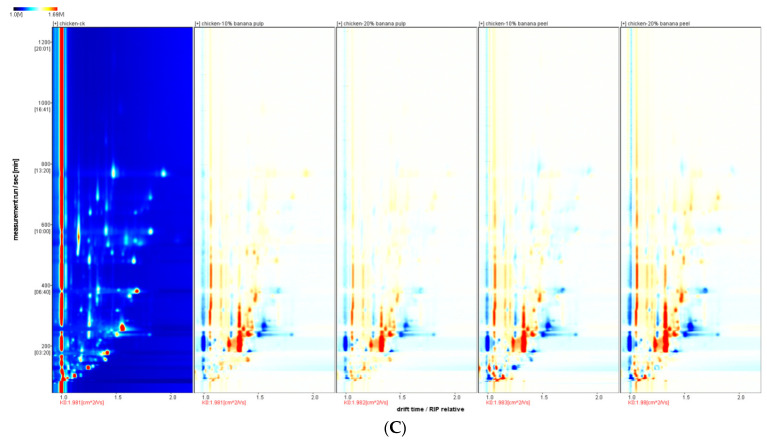
(**A**) 3D-topographic plots, (**B**) characteristic peak location point, and (**C**) 2D-topographic plots of the flavor substances in the chest meat samples of the experimental groups.

The identification and analysis of flavor compounds in the chest meat samples were conducted using HS-GC-IMS. The specific HS-GC-IMS analytical parameters are presented in Table 6. A total of 60 distinct flavor compounds were identified in the meat samples. The compounds were identified using the GC-IMS library, which includes 22 aldehydes, 14 alcohols, six ketones, two acids, one furan, and 15 compounds for which the functional group is currently undefined. The data are presented in Figure 1 as the topographical plots. The *x*-axis represents the ion migration time, and the *y*-axis represents the GC retention time. The peak intensity is represented on the *Z*-axis. The three-dimensional spectra of gas–ion migration demonstrate the flavor composition of the meat samples (Figure 1A). The flavor compositions exhibited comparable patterns. The results demonstrate that 3-hydroxy-2-butanone was exclusively detected in the meat samples of the supplemented chicken group but not in those of the CK group.

Figure 1B,C illustrate the two-dimensional spectrum of the gas–ion migration of the meat samples. The reactive ion peaks in the plots were subsequently normalized. Each data point to the right of the reactive ion peak represents a flavor compound present in the meat samples. The red points indicate a higher signal intensity, whereas the white spots indicate a lower signal intensity. The signal intensities, thus, represent the concentrations of the aforementioned flavor compounds. The meat sample from the CK group was utilized as a reference point. The ratio of a flavor compound in the meat samples of the supplementary group was identical to the reference ratio, which is indicated by a white spot after the deduction. The red and blue points indicate ratios that exceeded the reference value.

As illustrated in Figure 1C, the majority of the signals were observed within the retention time range of 50 and 800 s and the drift time range of 1.0 to 1.8. The flavor compounds, such as 2-ethylhexanol, exhibited notable differences between the various groups. Table 6 presents the qualitative analytical results for flavor compounds in samples of the chest muscle. Aldehydes were identified as the most significant flavor compounds in chicken meat, given their low flavor threshold and capacity to influence the overall flavor profile [53]. Noleau and Toulemonde [54] demonstrated that the removal of aldehydes resulted in the loss of the distinctive flavor profile of chicken meat, with an aroma that was more similar to beef. The aforementioned aldehydes, including hexanal, octanal, heptanal, nonanal, and trans-2-pentenal, as well as benzaldehyde and trans-2-nonenal, have also been identified in previous studies on chicken meat samples [55,56]. The literature also indicates that the majority of aldehydes, including hexanal, octanal, octenal, nonanal, 2, 4-heptadienal, 2-heptenal, and heptanal, are derived from lipid oxidation reactions [57,58]. Hexanal is the most prevalent aldehyde compound in chicken. It is the primary product of the oxidation of linoleic acid [55]. Additionally, it possesses a subtle aroma reminiscent of grass [59].

In addition to aldehydes, alcohols and ketones are the other products of lipid metabolism [60]. The flavor thresholds of alcohol are relatively high, and their contribution to the flavor of chicken meat is relatively low. The flavor thresholds of ketones are lower than those of aldehydes, and their contribution to the flavor of the meat is also lower. Among the alcohols identified in the chicken meat, 1-octene-3-ol was identified as the flavor compound that affected the meat flavor [56]. It has been demonstrated in pertinent research that 1-octene-3-ol evinces a flavor profile analogous to that of mushrooms [61]. It imparts a delightful soup-like quality to the flavor profile. Additionally, ketones are regarded as a significant flavor component in meat products [62].

A number of biochemical pathways are involved in the formation of flavor substances, including the Maillard reaction, lipid oxidation, and thiamine degradation [63]. The Maillard reaction is a non-enzymatic browning process that produces a range of compounds, including pyridine, pyrrole, pyrazine, thiophene, thiazole, and furanone [55]. The degradation of lipids encompasses the oxidation and hydrolysis of esters, which give rise to a range of flavor compounds, including alcohols, aldehydes, ketones, esters, and furans [56,64].

Moreover, these lipid oxidation products can also interact with Maillard reaction products, resulting in the formation of new flavor compounds. Thiamine degradation refers to the breakdown of nitrogen- and sulfur-containing bicyclic compounds, which, in turn, yield a range of nitrogen-, sulfur-, and heterocyclic-containing compounds, including thiophenes, furans, and thiazoles.

### 3.5. Characteristic Fingerprint of Flavor Compounds in Chicken Chest Meats

**Figure 2:**
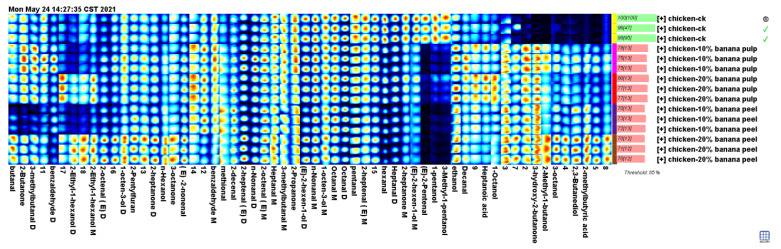
Fingerprint of flavor substances in the chest meat samples of the experimental groups.

To comprehensively analyze the differences in flavor composition of the chest meat samples, all flavor compound peaks identified in the spectrograms of these samples were selected to form fingerprints (Figure 2). The results demonstrated that the meat samples in the Pu-10, Pu-20, Pe-10, and Pe-20 groups exhibited the presence of specific flavor compounds that were not observed in other samples. These compounds include 3-hydroxy-2-butanone, 2-methylbutyric acid, 3-octanol, 2,3-butanediol, and 2-methyl-1-butanol. The meat samples in the Pe-20 group exhibited the highest levels of these flavor compounds. The levels of ethanol, 1-octanol, decanal, and heptanoic acid levels in the meat samples of the Pu-10, Pu-20, Pe-10, and Pe-20 groups were found to be higher than those observed in the control group. In contrast, the levels of pentanal, (E)-2-pentenal, hexanal, heptanal, octanal, 1-pentanol, 3-methyl-1-pentanol, and (E)-2-hexen-1-ol in the meat samples of the control group were higher than those observed in the supplementary groups. However, the levels of these flavor compounds in the meat samples of the supplementary groups exhibited variability.

The incorporation of varying quantities of the fermented banana samples into the poultry feeds resulted in notable alterations in the levels of flavor compounds present in the meat samples. Therefore, the presence of butanal, 3-methyl-butanal, (E)-2-heptenal, (E)-2-octenal, (E)-2-nonenal, N-nonanal, n-hexanol, 2-methyl-1-butanol, 2-ethyl-1-hexanol, 1-octen-3-ol, 3-octanol, 2,3-butanediol, 2-butanone, 2-heptanone, 3-octanone, 2-methylbutyric acid, and 2-pentylfuran was confirmed. The proportion of fermented banana peel added to the poultry feed was found to have a significant impact on the levels of certain flavor compounds present in the meat samples. However, the levels of certain other flavor compounds in the meat samples of the chickens fed with 20% fermented banana samples were found to be lower than in the chickens given 10% fermented banana samples.

### 3.6. Multivariate Analysis of Flavor Compounds in Chicken Chest Meats

**Figure 3:**
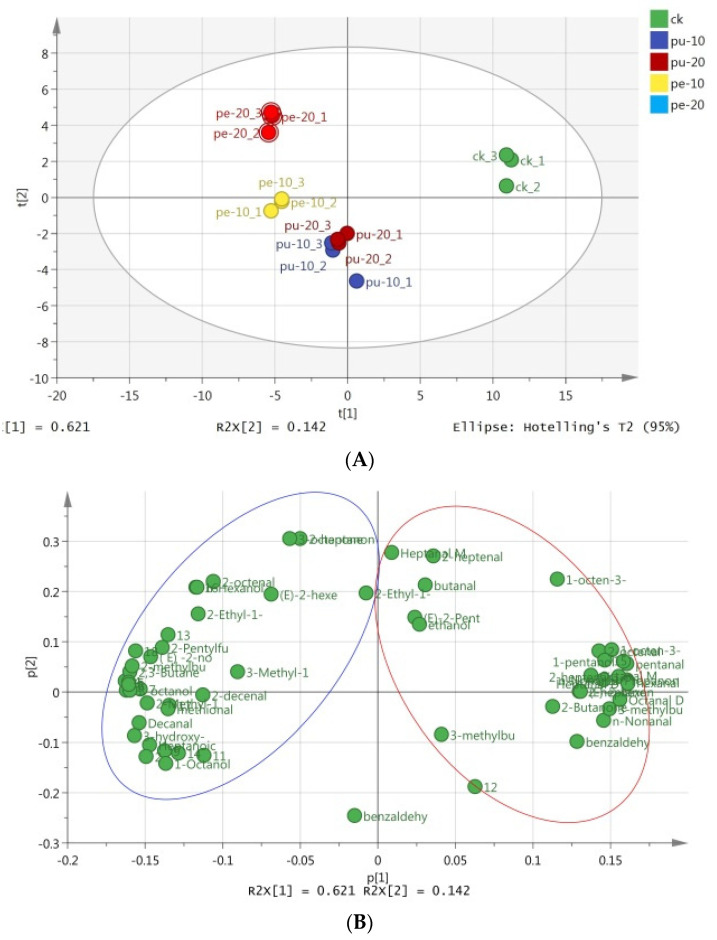
PCA (**A**) scores and (**B**) loading plot in the chest meat samples of the experimental groups.

Principal component analysis (PCA) represents one of the most commonly employed methods for reducing the dimensionality of data sets. The method was employed for the purpose of analyzing the principal chemical components present in the samples of chest meat. PCA reduces the number of multivariate data indicators to a smaller number, thereby facilitating comprehensive simplification of the multivariate statistical analysis [65]. As illustrated in Figure 3A, PC1 and PC2 collectively accounted for 62.1% and 14.2% of the total variance, respectively. The cumulative variance contribution rate of the two principal components was 75.3%. This result demonstrates that the two PCs collectively accounted for 75.3% of the total variance. The majority of the original information present in the two PCs was retained. Moreover, the analytical efficacy of the PCs was satisfactory.

The flavor profiles of the meat samples in the supplementary groups exhibited notable differences from those of the control group. Additionally, the flavor composition and levels of the meat samples in the Pu groups were found to be similar. As illustrated in Figure 3B, the aromatic substances displayed on the right side of the PCA loading plot exhibited a positive correlation with PC1. A positive PC1 score was observed for these compounds in the CK group. In contrast, the aromatic substances displayed on the left side of the PCA loading plot exhibited a negative correlation with PC1. This is evidenced by the negative PC1 scores observed for these flavor compounds in the Pe groups.

An OPLS-DA model was employed in the analysis to facilitate a deeper comprehension of the distribution patterns of these flavor compounds in the meat samples of the various experimental groups [66]. As a supervised multivariate statistical analysis method, OPLS-DA was characterized by the removal of data variation in the independent variable X, which was found to be unrelated to the categorical variable Y. Additionally, the information was analyzed as a single PC. Consequently, the model is straightforward and readily comprehensible. The discriminant and visualization effects of the PC score plot are well-established. In this study, the OPLS-DA model was employed to identify the most statistically significant variables based on the data matrix regression modeling of the flavor compound peaks detected in the chest meat samples. This was followed by the specific marker compounds that caused the difference in flavor being identified.

**Figure 4:**
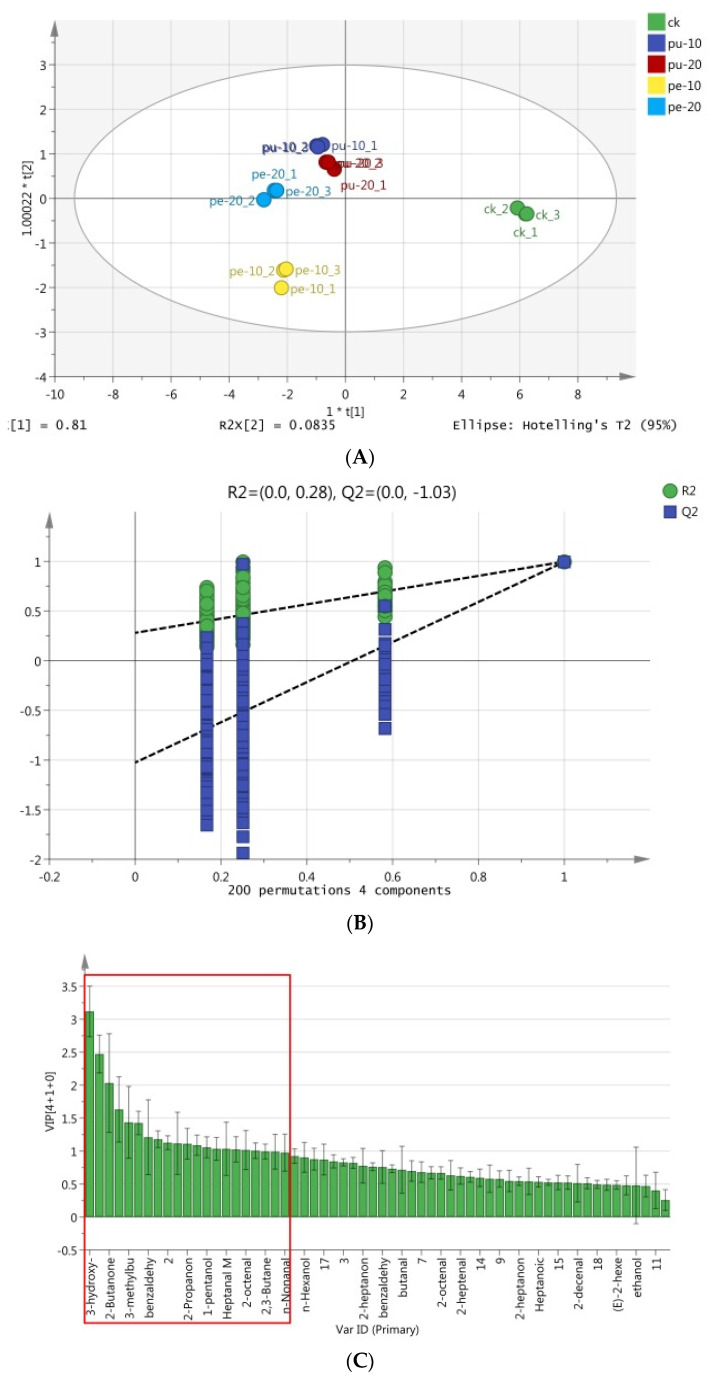
OPLS-DA of the flavor substances in the chest meat samples. (**A**) Scores, (**B**) permutation test, and (**C**) VIP plot based on the relative amounts of the substances. The flavor substances within the red box exhibited a VIP score greater than 1.

The peak information of flavor compounds in chest meat samples was initially standardized to remove the unit limitation of the data and then converted into a pure value without dimension. This approach allows for the lower peak index to be achieved without compromising the flavor value or undermining the reliability of the result. The results of the OPLS-DA analysis of these substances in the meat samples are presented in Figure 4. The total variance was 89.35%, the R2Y value was 99.1%, and the Q2 value was 94.2%. The flavor compounds in the meat samples were distributed across three of out the four quadrants, with the Pu-10, Pu-20, and Pe-20 groups situated in the second quadrant (with the exception of Pe-20-2), the Pe-10 group in the first quadrant, and the CK group in the fourth quadrant (Figure 4A). A permutation test was conducted as part of the analysis. Subsequently, the experimental data were randomly rearranged by modifying the sorting order of the categorical variable (Y), and Q2Y was randomly assigned up to 200 times to validate the model.

Figure 4B illustrates the outcomes of the permutation test result, wherein the regression line at the Q2 point is observed to intersect the vertical axis below zero, thereby indicating that the discriminant model did not exhibit signs of overfitting the data [67]. It can, thus, be concluded that the initial model was superior to the random arrangement model. The 18 characteristic flavor compounds in different groups were also selected according to the variable projection importance index (VIP), which exceeded 1 (Figure 4C). The identified substances were 3-hydroxy-2-butanone, hexanal, 2-butanone, 2-ethyl-1-hexanol M, 3-methylbutanal D, heptanal D, benzaldehyde D, and 2-methylbutyric acid. The remaining compounds were (E)-2,2-heptenal D, 2-propanone, pentanal, 1-pentanol, 1-octen-3-ol M, hepatanal M, 2-methyl-1-butanol, (E)-2-octenal D, 2,3-butanediol, and n-nonanal M.

### 3.7. Cluster Heat Map of Flavor Compounds in Chicken Chest Meats

**Figure 5:**
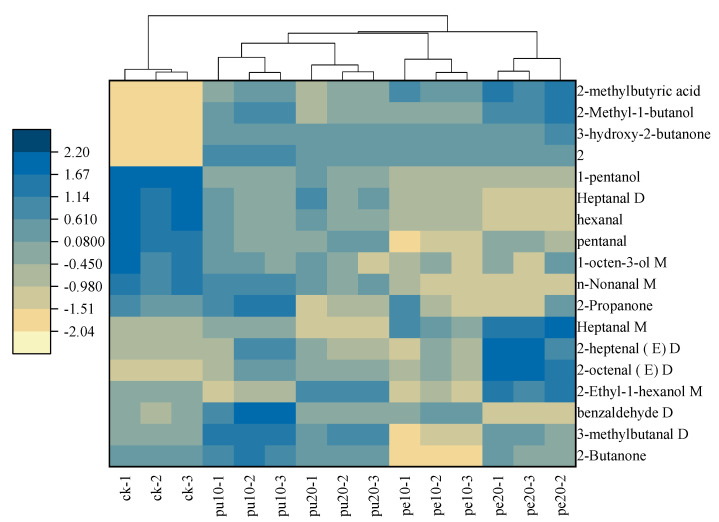
Cluster heat map of characteristic flavor substances in the chest meat samples of the experimental groups.

The OPLS-DA model was employed for the purpose of analyzing the characteristic flavor compounds present in the chest meat samples. A hierarchical cluster analysis was conducted on the 18 characteristic flavor compounds with a VIP score greater than 1. The heat map of these flavor substances was presented to illustrate the clustering of the flavor compounds (Figure 5). The light- to dark-colored squares of the heat map were used to indicate the relative levels of characteristic flavor compounds, with low levels represented by light squares and high levels represented by dark squares. The hierarchical cluster analysis (HCA) yielded two categories, the first of which included the control group (CK) and the second of which included the supplementary groups (Pu-10, Pu-20, Pe-10, and Pe-20).

The results demonstrate that the relative content of 2-methylbutyric acid, 2-methyl-1-butanol, 3-hydroxy-2-butanone, and compound 2 in the initial category was less than that observed in the subsequent category. However, the relative content of 1-pentanol, heptanal D, hexanal, pentanal, 1-octen-3-ol M, and N-nonanal M in the first category was higher than in the second category. Of these, hexanal and 1-octen-3-ol were identified as the key flavor compounds in the chest meat samples [56]. The observed differences in aroma among the meat samples were attributed to variations in flavor content.

The cluster heat map revealed that 2-methylbutyric acid, 2-methyl-1-butanol, 3-hydroxy-2-butanone, and compound 2 were the flavor compounds exclusively identified in the supplementation groups and not in the control group. Furthermore, (E)-2-octenal D was also a less prominent flavor compound in the CK group. In contrast, the distinctive flavor compounds identified in the control group’s meat sample were 1-pentanol, heptanal D, hexanal, pentanal, 1-octen-3-ol M, and N-nonanal M. However, the meat samples of the chickens fed with the diets containing fermented banana peels exhibited lower amounts of these flavor compounds.

The results demonstrate that the meat samples of the chickens fed with the diets supplemented with the fermented banana peels (Pe group) exhibited a reduced proportion of ketone and an increased proportion of alcohol in comparison to the Pu and control groups. Additionally, the proportion of benzaldehyde D was observed to be lower in the Pe-20 group than in the other experimental groups. The presence of this aromatic compound in the meat sample, given its bitter taste, results in a reduction in its quality. Therefore, the meat samples of the chickens fed with diets containing fermented banana peels, irrespective of the quantity added, exhibited superior quality in terms of physical and nutritional characteristics.

## 4. Conclusions

The incorporation of varying proportions of fermented banana peel and pulp residue into the daily feed regimen resulted in increased carcass weights and the percentages of chicken pectoral and thigh muscles, as evidenced by the statistical analysis. Moreover, improvements were observed in the moisture content, FAs, and flavor components of the chest meat samples. The mean weight gain of the experimental chickens was 2.51 kg and 2.83 kg, with a pronounced increase in pectoral and thigh muscles observed in the Pe group in comparison to the control group. The supplementation groups demonstrated a greater degree of redness, tenderness, and moisture content in their chest meat samples when compared to the control group. Furthermore, the supplemented groups demonstrated a higher total FA content than the control group. The predominant SFAs in the chest meat samples were palmitic acid and stearic acid, while the principal MUFAs were palmitoleic acid and oleic acid, with linoleic acid representing the primary PUFA. Additionally, the meat samples exhibited the presence of 3-hydroxy-2-butanone, 2-methylbutyric acid, 3-octanol, 2,3-butanediol, and 2-methyl-1-butanol, which were identified as unique flavor components in comparison to the control sample. Furthermore, the hierarchical cluster analysis revealed 18 distinctive characteristic flavor compounds with VIP scores exceeding 1. In conclusion, the incorporation of varying proportions of fermented banana products, exclusively the fermented banana peels, into the daily diet could enhance the quality of chicken and influence its flavor profile. The data may also be employed as a theoretical foundation for the utilization of banana by-products as premium animal feed.

## Figures and Tables

**Table 1:** Chemical constituents of the fermented banana peel and pulp residue.

Sample	Ash (%)	DM (%)	pH	CHO (%)	CP (%)	Titratable Acid (%)	LAB (log CFU)
Banana peel	13.62 ± 0.17 ^a^	9.16 ± 0.06 ^b^	4.60 ^a^	0.02 ± 0.00 ^b^	5.77 ± 0.01 ^b^	11.59 ± 0.03 ^b^	8.48 ± 0.07 ^a^
Pulp residue	8.59 ± 0.96 ^b^	25.69 ± 0.66 ^a^	3.51 ^b^	0.45 ± 0.09 ^a^	10.28 ± 0.10 ^a^	14.18 ± 0.31 ^a^	3.51 ± 0.02 ^b^

The presence of different superscript lowercase letters (^a,b^) within the same column indicates a statistically significant difference between the two groups (*p* < 0.05). DM, dry matter; CHO, carbohydrates; CP, crude protein; LAB, lactic acid bacteria.

**Table 2:** Poultry diet composition.

Group	Percentage				
	CK	Pe-10	Pe-20	Pu-10	Pu-20
Basal diet	100	90	80	90	80
Banana peel	0	10	20	0	0
Banana pulp residue	0	0	0	10	20

The basal diet consisted of a mixture of the following ingredients: 57.5% corn, 25% soybean meal, 5% wheat bran, 2.5% soy oil, and 10% premix.

**Table 3:** Effects of incorporating varying proportions of fermented banana samples on the slaughter performance of the experimental chickens.

Group	Slaughter Performance				
	Carcass Weight (kg)	Net Weight (kg)	Abdominal Fat (g)	Pectoral Muscle (%)	Leg Muscle (%)
CK	3.76 ± 0.19 ^b^	3.18 ± 0.13 ^a^	158.92 ± 24.04 ^ab^	13.55 ± 1.67 ^c^	13.45 ± 1.11 ^b^
Pe-10	3.76 ± 0.27 ^b^	3.19 ± 0.28 ^a^	158.27 ± 43.15 ^ab^	14.77 ± 1.81 ^b^	16.89 ± 2.51 ^a^
Pe-20	3.95 ± 0.17 ^ab^	3.33 ± 0.10 ^a^	158.97 ± 63.27 ^ab^	17.22 ± 2.08 ^a^	17.31 ± 1.97 ^a^
Pu-10	4.08 ± 0.24 ^a^	3.39 ± 0.22 ^a^	117.02 ± 25.08 ^b^	15.83 ± 1.88 ^ab^	15.50 ± 1.82 ^ab^
Pu-20	3.82 ± 0.36 ^ab^	3.19 ± 0.26 ^a^	197.22 ± 23.68 ^a^	14.65 ± 1.02 ^bc^	15.50 ± 1.49 ^ab^

The presence of different superscript lowercase letters (^a–c^) in the same column indicates a statistically significant difference between the treatment groups (*p* < 0.05). The net weight of a chicken is defined as the weight of the carcass without the feathers.

**Table 4:** Effects of adding different proportions of fermented banana samples on the quality characteristics of the chest meat samples.

Index of Quality	CK	Pe-10	Pe-20	Pu-10	Pu-20
Color parameters					
L*	62.08 ± 0.93 ^b^	60.76 ± 1.52 ^bc^	62.58 ± 0.73 ^b^	58.04 ± 2.04 ^c^	66.79 ± 0.33 ^a^
a*	3.06 ± 0.41 ^c^	4.89 ± 0.75 ^a^	4.41 ± 0.48 ^b^	4.55 ± 0.46 ^ab^	3.09 ± 0.75 ^c^
b*	5.33 ± 0.66 ^b^	2.63 ± 0.78 ^d^	5.24 ± 0.87 ^b^	7.21 ± 0.34 ^a^	3.71 ± 0.22 ^c^
Shear force	17.86 ± 0.50 ^a^	10.61 ± 1.06 ^b^	14.20 ± 1.8 ^b^	13.44 ± 0.98 ^b^	15.49 ± 0.32 ^b^
Moisture	71.02 ± 0.11 ^a^	71.03 ± 0.12 ^a^	71.27 ± 0.44 ^a^	70.64 ± 0.48 ^b^	69.82 ± 0.44 ^c^
Water-holding capacity	46.39 ± 0.40 ^c^	48.91 ± 0.82 ^a^	48.31 ± 0.31 ^ab^	48.75 ± 0.87 ^b^	47.46 ± 0.74 ^b^

The presence of different superscript lowercase letters (^a–d^) within the same line indicates a statistically significant difference between the treatment groups (*p* < 0.05).

**Table 5:** Effect of different proportions of fermented banana samples on fatty acids in the chest meats of the experimental chickens.

Item	Fatty Acid Content (mg/100 g)				
	CK	Pe-10	Pe-20	Pu-10	Pu-20
C8:0	-	3.3 ± 0.1	3.1 ± 0.2	-	-
C10:0	-	3.1 ± 0.1	3.0 ± 0.1	-	-
C11:0	-	-	3.1 ± 0.3	-	-
C13:0	-	-	2.1 ± 0.1	-	-
C14:0	7.7 ± 0.1	14.2 ± 0.2	13.6 ± 0.1	9.4 ± 0.2	13.0 ± 0.4
C14:1	-	2.8 ± 0.1	2.4 ± 0.0	-	-
C15:0	-	2.2 ± 0.1	-	-	-
C16:0	333.0 ± 4.6	552.3 ± 8.4	543.9 ± 0.5	392.1 ± 3.7	502.7 ± 13.7
C16:1	41.4 ± 0.8	72.5 ± 1.0	71.7 ± 0.2	46.8 ± 0.6	70.6 ± 1.8
C17:0	-	2.5 ± 0.2	3.0 ± 0.2	2.4 ± 0.1	2.8 ± 0.0
C17:1	-	2.5 ± 0.0	1.9 ± 0.0	-	-
C18:0	120.9 ± 2.1	176.0 ± 3.3	180.9 ± 0.2	127.9 ± 1.2	163.4 ± 4.6
C18:1n9t	8.3 ± 0.3	11.2 ± 0.5	10.6 ± 0.0	6.7 ± 0.0	10.1 ± 0.3
C18:1n9c	363.4 ± 4.8	680.4 ± 11.1	682.3 ± 0.2	492.2 ± 4.5	632.7 ± 17.2
C18:2n6t	4.3 ± 0.1	-	-	-	-
C18:2n6c	125.7 ± 1.8	195.2 ± 4.0	232.3 ± 0.4	173.8 ± 1.8	245.3 ± 4.5
C20:0	-	-	4.0 ± 0.0	-	-
C18:3n6	-	-	2.8 ± 0.0	-	3.1 ± 0.1
C18:3n3	5.5 ± 0.0	9.4 ± 0.3	9.6 ± 0.0	9.1 ± 0.2	14.1 ± 0.2
C20:2	-	-	2.4 ± 0.1		2.4 ± 0.1
C20:3n6	7.9 ± 0.0	11.6 ± 0.2	10.8 ± 0.3	7.1 ± 0.3	10.1 ± 0.0
C20:4n6	14.1 ± 0.3	23.5 ± 0.0	22.9 ± 0.2	13.1 ± 0.1	18.8 ± 0.5
C22:0	-	-	-	-	2.7 ± 0.1
C23:0	4.9 ± 0.1	3.9 ± 0.1	2.8 ± 0.2	2.4 ± 0.2	-
C24:1	-	2.8 ± 0.1	-	3.3 ± 0.1	-
C22:6n3	-	4.4 ± 0.2	3.5 ± 0.1	-	3.1 ± 0.1
Total FAs	1038 ± 14 ^b^	1774 ± 29 ^a^	1813 ± 1 ^a^	1286 ± 12 ^b^	1695 ± 43 ^a^
Total SFAs	467 ± 7 ^b^	757 ± 13 ^a^	760 ± 1 ^a^	534 ± 5 ^a^	684 ± 19 ^a^
Total MUFAs	413 ± 5 ^b^	772 ± 12 ^a^	769 ± 0.0 ^a^	549 ± 5 ^a^	714 ± 19 ^a^
Total PUFAs	158 ± 2 ^b^	245 ± 4 ^a^	284 ± 0.0 ^a^	203 ± 1 ^a^	297 ± 5 ^a^

The presence of different superscript lowercase letters (^a,b^) within the same line indicates a statistically significant difference between the treatment groups (*p* < 0.05). FAs, fatty acids; SFAs, saturated fatty acids; MUFAs, monounsaturated fatty acids; PUFAs, polyunsaturated fatty acids.

**Table 6:** HS-GC-IMS integration parameters of volatile compounds in the chest meats of the experimental chickens.

	Compound	CAS	Formula	RI	Rt [s]	Dt [a.u.]
1	Ethanol	C64175	C_2_H_6_O	507.4	96.283	1.05095
2	2-Propanone	C67641	C_3_H_6_O	522.7	102.745	1.12059
3	2-Butanone	C78933	C_4_H_8_O	589.4	130.731	1.24919
4	Butanal	C123728	C_4_H_8_O	597.6	134.19	1.29085
5	3-Methylbutanal M	C590863	C_5_H_10_O	644.2	153.755	1.17545
6	3-Methylbutanal D	C590863	C_5_H_10_O	654.2	157.948	1.40341
7	Pentanal	C110623	C_5_H_10_O	694.3	177.022	1.41981
8	2-Methylpropanoic acid	C79312	C_4_H_8_O_2_	703.4	184.309	1.15804
9	3-Hydroxy-2- 4-butanone	C513860	C_4_H_8_O_2_	705	185.618	1.33006
10	2-Methyl-1-butanol	C137326	C_5_H_12_O	729.8	205.655	1.23342
11	(E)-2-pentenal	C1576870	C_5_H_8_O	749.6	221.551	1.10733
12	1-Pentanol	C71410	C_5_H_12_O	770.3	238.233	1.50366
13	2	UC	UC	770.4	238.307	1.41003
14	3	UC	UC	789.3	254.983	1.44704
15	2,3-Butanediol	C513859	C_4_H_10_O_2_	791.3	257.174	1.36463
16	Hexanal	C66251	C_6_H_12_O	793.1	259.061	1.55972
17	4	UC	UC	813	280.583	1.4403
18	3-Methyl-1- 4-pentanol	C589355	C_6_H_14_O	816	283.731	1.59823
19	(E)-2-hexen-1-ol M	C928950	C_6_H_12_O	850.7	321.182	1.17871
20	(E)-2-hexen-1-ol D	C928950	C_6_H_12_O	847.6	317.872	1.51793
21	5	UC	UC	847.9	318.218	1.42165
22	n-Hexanol	C111273	C_6_H_14_O	883.5	356.538	1.325
23	2-Heptanone M	C110430	C_7_H_14_O	892.6	366.767	1.25942
24	2-Heptanone D	C110430	C_7_H_14_O	893.4	368.142	1.63053
25	Heptanal M	C111717	C_7_H_14_O	901.1	381.665	1.3368
26	Heptanal D	C111717	C_7_H_14_O	900.5	380.666	1.69431
27	7	UC	UC	902.9	384.915	1.52391
28	Methional	C3268493	C_4_H_8_OS	919.7	414.656	1.08895
29	8	UC	UC	944.7	458.843	1.47603
30	(E)-2-heptenal M	C18829555	C_7_H_12_O	958.6	483.399	1.25555
31	(E)-2-heptenal D	C18829555	C_7_H_12_O	957.2	480.916	1.66598
32	9	UC	UC	958.6	483.296	1.38048
33	3-Octanol	C589980	C_8_H_18_O	972	506.958	1.39864
34	Benzaldehyde M	C100527	C_7_H_6_O	973.5	509.644	1.15064
35	Benzaldehyde D	C100527	C_7_H_6_O	973.2	509.143	1.46535
36	11	UC	UC	970.2	503.781	1.56608
37	12	UC	UC	987.7	534.732	1.42819
38	3-Octanone	C106683	C_8_H_16_O	989.5	537.863	1.72064
39	2-Pentylfuran	C3777693	C_9_H_14_O	994.3	546.475	1.25261
40	13	UC	UC	996.1	549.566	1.67793
41	1-Octen-3-ol M	C3391864	C_8_H_16_O	997	551.265	1.1506
42	1-Octen-3-ol D	C3391864	C_8_H_16_O	996.6	550.484	1.59549
43	14	UC	UC	1008.6	574.563	1.46223
44	Octanal M	C124130	C_8_H_16_O	1010.6	578.601	1.41146
45	Octanal D	C124130	C_8_H_16_O	1011.1	579.6	1.82154
46	15	UC	UC	1041	639.359	1.26226
47	Heptanoic acid	C111148	C_7_H_14_O_2_	1041.7	640.785	1.36674
48	16	UC	UC	1042	641.363	1.67619
49	17	UC	UC	1048.6	654.572	1.45112
50	18	UC	UC	1045.1	647.503	1.73727
51	2-Ethyl-1- 3-hexanol M	C104767	C_8_H_18_O	1049.5	656.388	1.41109
52	2-Ethyl-1- 3-hexanol D	C104767	C_8_H_18_O	1051	659.393	1.79091
53	1-Octanol	C111875	C_8_H_18_O	1063.9	685.128	1.45646
54	(E)-2-octenal M	C2548870	C_8_H_14_O	1067.7	692.79	1.33051
55	(E)-2-octenal D	C2548870	C_8_H_14_O	1067.3	691.963	1.81216
56	n-Nonanal M	C124196	C_9_H_18_O	1105.4	768.275	1.47591
57	n-Nonanal D	C124196	C_9_H_18_O	1105.4	768.275	1.93479
58	(E)-2-nonenal	C18829566	C_9_H_16_O	1159.9	877.417	1.40888
59	Decanal	C112312	C_10_H_20_O	1210.7	978.942	1.53654
60	2-Decenal	C3913711	C_10_H_18_O	1292.4	1142.437	1.47965

RI, Retention index; Rt, Retention time; Dt, Drift time; UC, Undefined compound.

## Data Availability

The original contributions presented in the study are included in the article, further inquiries can be directed to the corresponding author.
